# Precise A•T to G•C base editing in the zebrafish genome

**DOI:** 10.1186/s12915-018-0609-1

**Published:** 2018-11-20

**Authors:** Wei Qin, Xiaochan Lu, Yunxing Liu, Haipeng Bai, Song Li, Shuo Lin

**Affiliations:** 1 State Key Laboratory of Chemical Oncogenomics, Key Laboratory of Chemical Genomics, Peking University Shenzhen Graduate School, Shenzhen, 518055 China; 2 Department of Molecular, Cell and Developmental Biology, University of California, Los Angeles, CA 90095 USA

**Keywords:** Adenine base editors, Zebrafish, Base editing

## Abstract

**Background:**

Base editors are a class of genome editing tools with the ability to efficiently induce point mutations in genomic DNA, without inducing double-strand breaks or relying on homology-direct repair as in other such technologies. Recently, adenine base editors (ABEs) have been developed to mediate the conversion of A•T to G•C in genomic DNA of human cells, mice, and plants. Here, we investigated the activity and efficiency of several adenine base editors in zebrafish and showed that base editing can be used to create new models of pathogenic diseases caused by point mutations.

**Results:**

The original ABE7.10 exhibits almost no activity in zebrafish. After codon optimization, we found that a zABE7.10 variant could induce targeted conversion of adenine to guanine in zebrafish at multiple tested genomic loci, and all the target sites showed a high rate of germline targeting efficiency. Furthermore, using this system, we established a zebrafish model of 5q-Syndrome that contained a new point mutation in *rps14*. The further modification of zABE7.10 by a bipartite nuclear localization signals (bpNLS) resulted in 1.96-fold average improvement in ABE-mediated editing efficiency at four sites.

**Conclusions:**

Collectively, this system, designated as zABE7.10, provides a strategy to perform A•T to G•C base editing in zebrafish and enhances its capacity to model human diseases.

**Electronic supplementary material:**

The online version of this article (10.1186/s12915-018-0609-1) contains supplementary material, which is available to authorized users.

## Background

Clinical studies have shown that many genetic disorders are caused by genomic point mutations affecting single amino acids instead of whole gene disruption. Although it is possible to model these point mutations by gene editing through homology-directed recombination (HDR), the efficiency of establishing precise mutations in animals remains low [1]. In order to complement the HDR method, one approach called “base editing” was developed, which can irreversibly convert C to T at target loci without double-strand break (DSB) [2, 3]. Among these base editors, the third-generation base editor (BE3) has been applied in several species, including zebrafish [4–6]. To further improve these base editors, strategies have been developed to expand editing window [7], increase editing specificity and efficiency [8, 9], improve base excision repair inhibition [10], and change protospacer-adjacent motif (PAM) compatibilities [7]. However, these base editors only mediate the conversion of C to T, which reduces its application in gene editing.

Recently, using protein evolution, Gaudelli et al. successfully evolved an adenine deaminase and, based upon it, engineered several ABEs that can mediate the conversion of A to G in human cells. Among them, ABE7.10 can convert target A to G efficiently (~ 50% in human cells) with very high product purity (typically > 99.9%) and very low rates of indels (typically < 0.1%) [11]. More recently, this method has been applied successfully in plants, mouse, and human cells [12–14]. In this study, we further demonstrate that an optimized ABE system can efficiently generate A to G mutations in zebrafish.

## Results

### Codon-optimized zABE7.10 could induce base editing in zebrafish

**Fig. 1:**
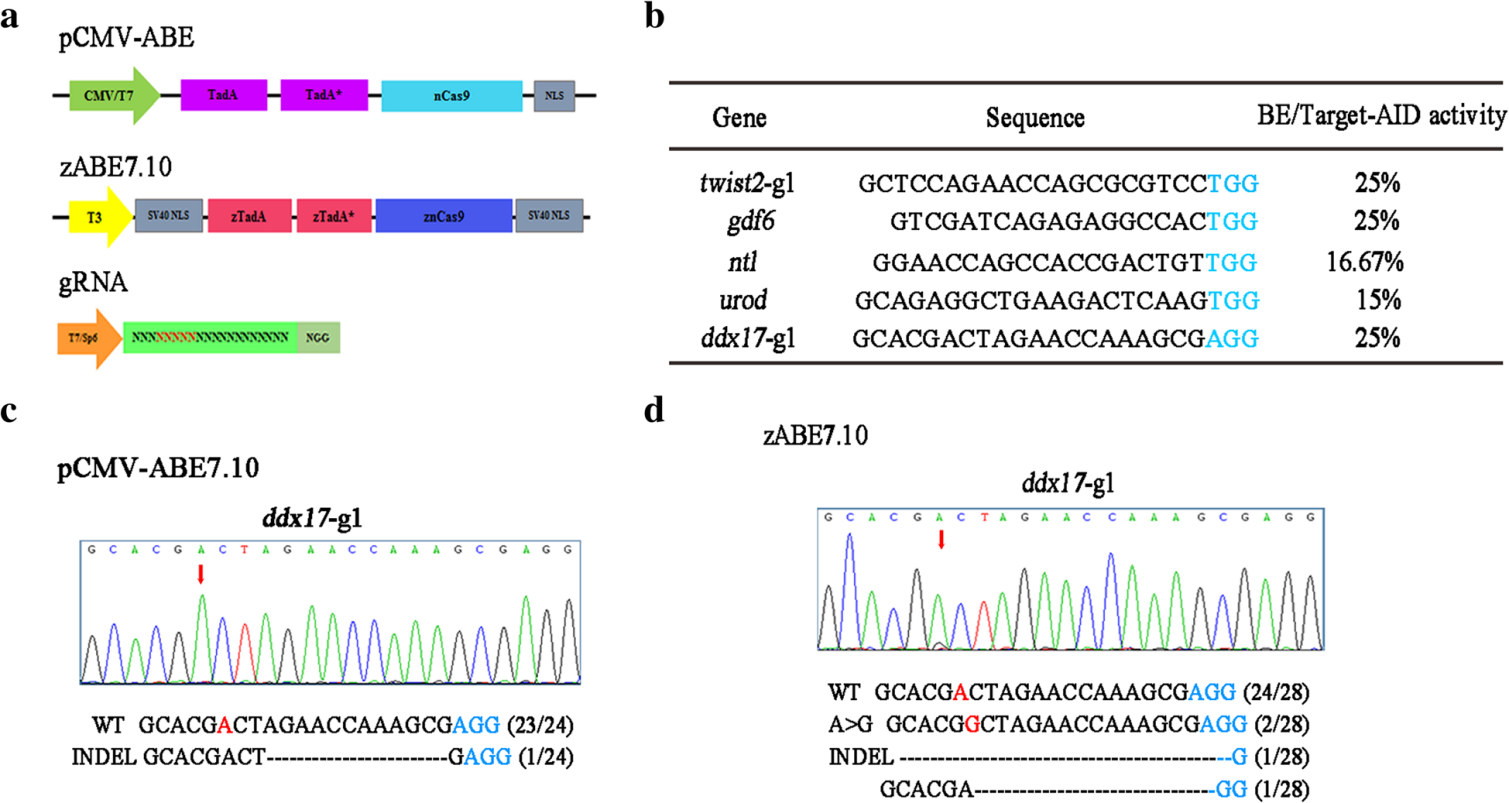
Comparison of the original ABE system and optimized zABE7.10 system in zebrafish. **a** Schematic diagrams of two adenine base-editing system, pCMV-ABE and zABE7.10. The zABE7.10 system is a zebrafish codon optimized version by IGE. **b** Selected sites that can work efficiently in zebrafish using BE system or Target-AID system. Target sequence (*black*) and PAM region (*blue*) are indicated. **c** Sequencing result at the *ddx17*-g1 target in pCMV-ABE system in zebrafish. **d** Sequencing result at the *ddx17*-g1 target in zABE7.10 system in zebrafish

To explore whether the pCMV-ABE7.10-gRNA complex can mediate A to G conversion in zebrafish genome (Fig. 1a), we selected gRNAs for five target sites (*twist2-*g1, *gdf6*, *ntl*, *urod*, and *ddx17*-g1) that have been previously shown to be efficient in zebrafish using BE or Target-AID system (Fig. 1b) [5, 15]. After injecting pCMV-ABE7.10 mRNA and related gRNA into one-cell embryos of zebrafish, six embryos at 48 h post fertilization (hpf) were randomly selected for genomic DNA extraction. PCR amplification of the region covering the target site was performed, and the products were analyzed by sequencing. Sequencing analysis indicated that the pCMV-ABE7.10 system did not induce base conversion at any of the selected loci (Fig. 1c).

We hypothesized that ABE might need some specific modifications to make it work in zebrafish. We synthesized zebrafish codon-optimized wild-type ecTadA and its mutant form ecTadA*7.10, and linked them to zCas9 using a 32-amino-acid linker in the pT3TS-nCas9n vector. We designated this new construct as zABE7.10 (Fig. 1a) and performed the same gene editing experiments as described above. Compared to the original system, sequencing PCR products of the target sites showed successful conversion of A to G by our optimized ABE system at the targeted adenine of *ddx17*-g1 locus. T-A cloning of PCR products followed by sequencing showed that 2 of the 28 colonies carried A → G conversion at site 6, 2 colonies had indels, and the remaining 24 maintained wild-type sequence (Fig. 1d). However, under the condition, overlapping sequencing signal peaks could not be directly detected at the other four sites. We next tested additional 23 genomic sites with different cleavage activities mediated by gRNAs (Additional file 1: Table S1). Among them, the conversion of A → G at 4 sites (*atp5b, rps14, wu:fc01d11*, *musk*) with frequency of 8.30–22.22% were obtained. This finding suggests that, although zABE 7.10 system can induce A → G base editing in zebrafish, it may have sequence preference over different target sites.

### The nucleotide substitution at target adenine of *rps14* recapitulate typical mutant phenotypes

**Fig. 2:**
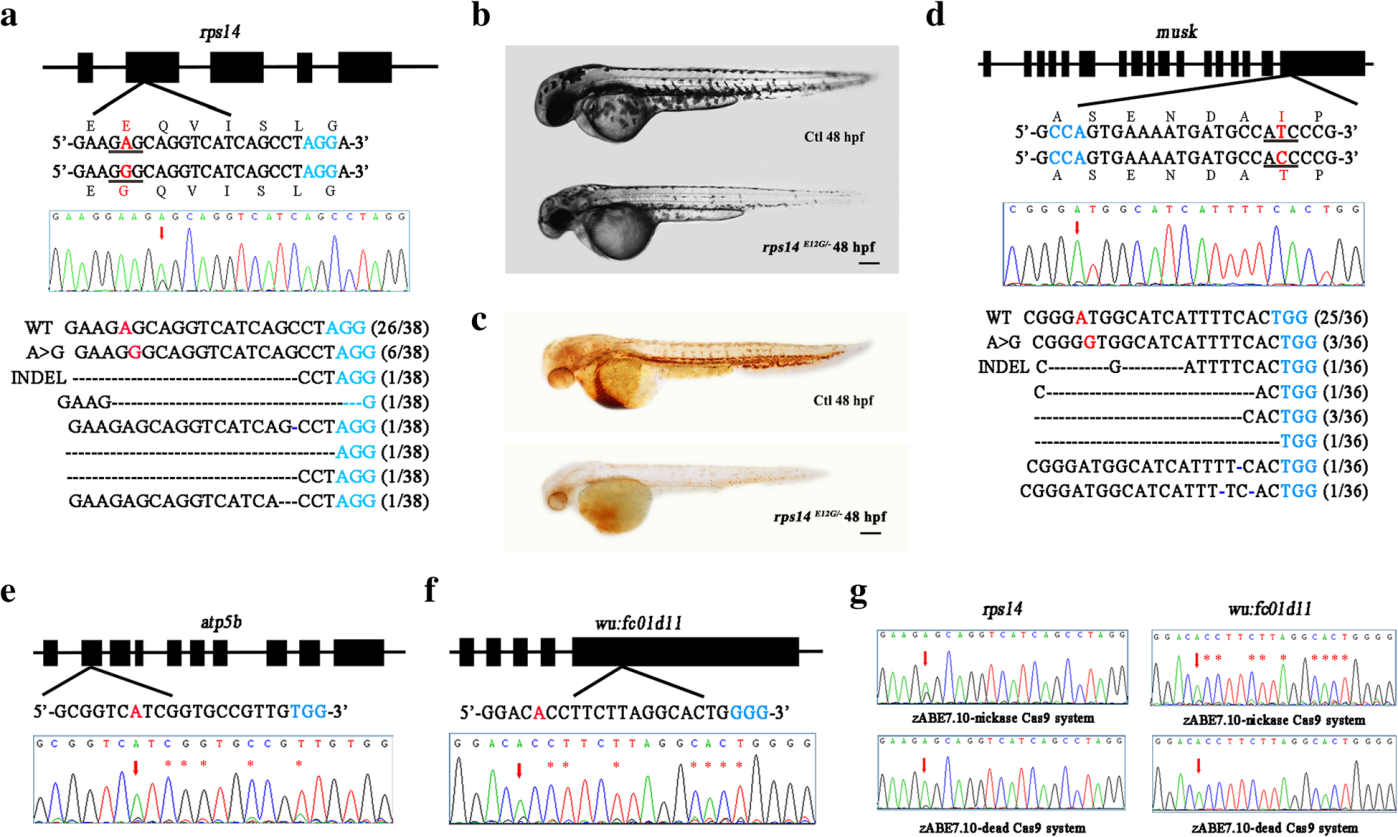
Codon-optimized zABE7.10 system in zebrafish induces A to G base conversion in zebrafish. **a** Schematic view of the gRNA target site in *rps14* gene and the sequencing results. **b** Morphological phenotype of *rps14* ^*E12G/−*^ deficient embryos. **c** *o*-dianisidine staining results of the *rps14* ^*E12G/−*^ embryos. **d** Schematic view of the gRNA target site in *musk* gene and the sequencing results. **e** Sequence chromatograms of *atp5b* locus. **f** Sequence chromatograms of *wu:fc01d11* locus. **g** Comparison the base editing efficiency between zABE7.10-nickcase Cas9 and zABE7.10-dCas9 system. Target sequence (*black*), PAM region (*blue*), and the substituted bases (*red*) are indicated. The black line presents the changed amino acid. The red arrows indicate the overlapped peaks. The black dashes and blue dashes denote deleted bases and inserted bases in the sequence, respectively. The red asterisks indicated the overlapping peaks outside the targeted A

For *rps14*, a candidate gene for 5q-syndrome [16], we generated a pathogenic point mutation E12G (Fig. 2a). When F0 founders mated to a previously generated *rps14* heterozygous mutant adult, several embryos exhibited a small head and small eyes at 2dpf (days post fertilization) similar to the *rps14*^*−/−*^ mutants (Fig. 2b). As expected, *o*-dianisidine staining of these embryos showed a decreased level of hemoglobin, suggesting a defect in the production of erythroid in these embryos [16] (Fig. 2c). Further genotyping confirmed that these embryos contained E12G encoded by A → G conversion.

### Generation of F0 adult zebrafish carrying the expected nucleotide substitutions

Congenital myasthenic syndrome (CMS) is an inherited neuromuscular disorder caused by defects of several types at the neuromuscular junction. Mutations in *MUSK* gene have been associated with this disease [17]. Human ClinVar database reported an I764T variant in the MUSK gene, but this variant was not observed in approximately 6000 normal individuals of European and African American ancestry in the NHLBI Exome Sequencing Project, suggesting that this mutation might be a causing mutation for CMS. This mutation is located within the protein kinase domain, at a position that is conserved across species. In silico analysis predicts that this variant should impair the protein structure/function, so that I764T variant is likely a pathogenic variant. To address this issue, we injected zABE7.10 mRNA and *musk* gRNA into zebrafish embryos and assessed the base conversion. For individual larvae, the overlapping peaks at the targeted A could be detected in each groups of randomly selected embryos (*n* = 12). TA cloning/sequencing showed 3 of 36 colonies carried A to G base conversion, indicating a target mutation frequency of 8.30% (Fig. 2d). It has been reported that homozygous null *musk*^−/−^ mutant zebrafish embryos have abnormal morphology and reduced outgrowth of ventrally projecting primary motor neurons [18]. We have obtained germline transmission of this mutation and should be able to determine if the I764T variant is pathogenic once we can mate the F1 fish when they reach sexual maturity.

### Evaluation of the zABE7.10 system in zebrafish embryos

**Fig. 3:**
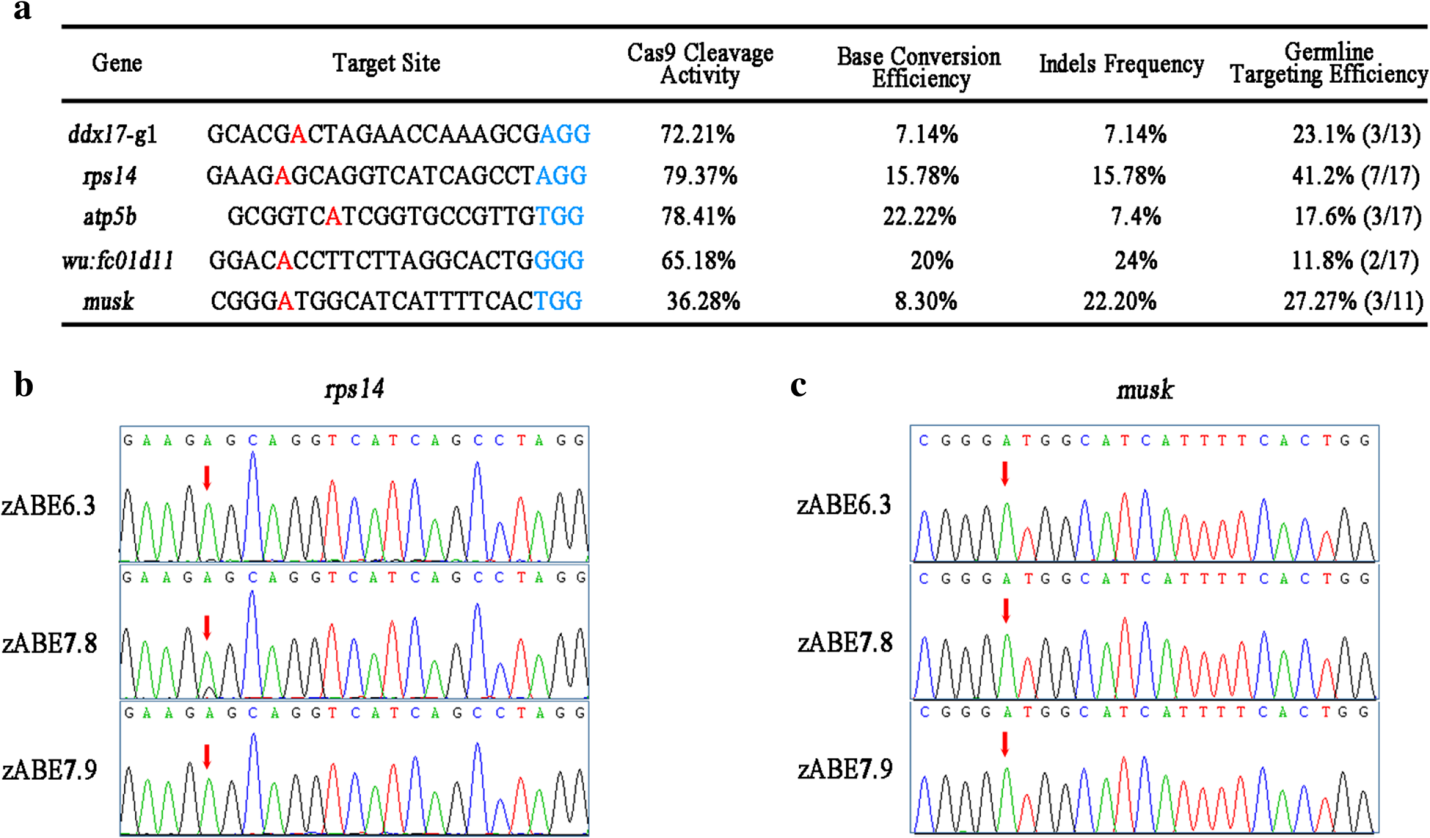
Summary of adenine base-editing results in zebrafish. **a** The base conversion efficiency, cleavage activities, indels frequency, and germline-targeting efficiency of five target sites (*ddx17*-g1, *rps14*, *atp5b*, *wu:fc01d11* and *musk*) are summarized. **b**, **c** The sequence chromatograms results of zABE6.3, zABE7.8, and zABE7.9 in the *rps14* and *musk* target site. Target sequence (*black*), PAM region (*blue*), and the substituted bases (*red*) are indicated. The red arrows indicate the overlapped peaks

Sequencing PCR products revealed that there were smaller but detectable overlap peaks outside the targeted adenine (Fig. 2e, f and Additional file 1: Figure S1), indicating possible indels. Analysis of individual products by TA cloning/sequencing for these sites revealed frequency of indels of 7.14–22.20%. We previously showed that replacing nCas9 (nickase Cas9) in BE system with dead Cas9 (dCas9) reduced indel formation in zebrafish [5]. So we constructed zABE7.10-dCas9 system and tested in zebrafish. We found that zABE7.10-dCas9 indeed reduced indel formation but also significantly reduced the A → G conversion at *rps14* and *wu:fc01d11* sites (Fig. 2g). Since authentic A → G conversion without indels can be easily identified through screening F1 germline transmission of nCas9 zABE7.10 system, we feel it is more desirable to use nCas9 than dCas9. Germline targeting rate is most important for evaluating gene-editing methods. We found that zABE7.10 exhibited a high rate of germline targeting efficiency at the five sites tested (Fig. 3a). We also randomly selected one positive F0 founder from each site and analyzed the germline transmission rate. Both targeted nucleotide substitutions and indels were heritable and germline transmission rate ranged from 25 to 58% (Additional file 1: Table S2).

We noted that zABE7.10 system had failed to induce A → G conversion for a number of tested loci in zebrafish. To address if this was caused by variations of Cas9 cleavage activities associated with different gRNAs, we analyzed the correlation of base conversion efficiency, cleavage activities, indels frequency, and germline-targeting efficiency for these sites (Fig. 3a). We found that efficiency of A → G conversion by zABE7.10 had no apparent correlation with its site-specific activity of Cas9 cleavage. For instance, no A → G conversion was observed at sites of *nop56, slc40a1*-s1 that had nearly 100% cleavage activity by Cas9/gRNA (Additional file 1: Table S1).

ABE6.3, ABE7.8, and ABE7.9 have been reported with slightly decreased deaminase activity and broadened editing window in human cells [11]. Next, we examined the efficiency of these tools by targeting *rps14* and *musk* loci in zebrafish. Only zebrafish codon-optimized ABE6.3 and ABE7.8 had minimal base conversions at *rps14* locus (Fig. 3b, c). Moreover, the candidate A at protospacer position 8 could not be targeted. These data suggested that zABE6.3, zABE7.8, and zABE7.9 had poor performance at two loci tested here.

To assess the potential off-target effects of zABE7.10 in zebrafish, we selected several off-target sites with up to 4-nucleotide mismatches at the non-seed region in its genome using Cas-OFFinder [19]. Sequencing analysis suggested that there were no off-target conversion of A → G conversion at these sites (Additional file 1: Figure S2). These results demonstrated that zABE7.10 is a highly specific programmable tool for targeted base editing in zebrafish.

### Modification of nuclear localization signals further improves the base editing ability in zebrafish

**Fig. 4:**
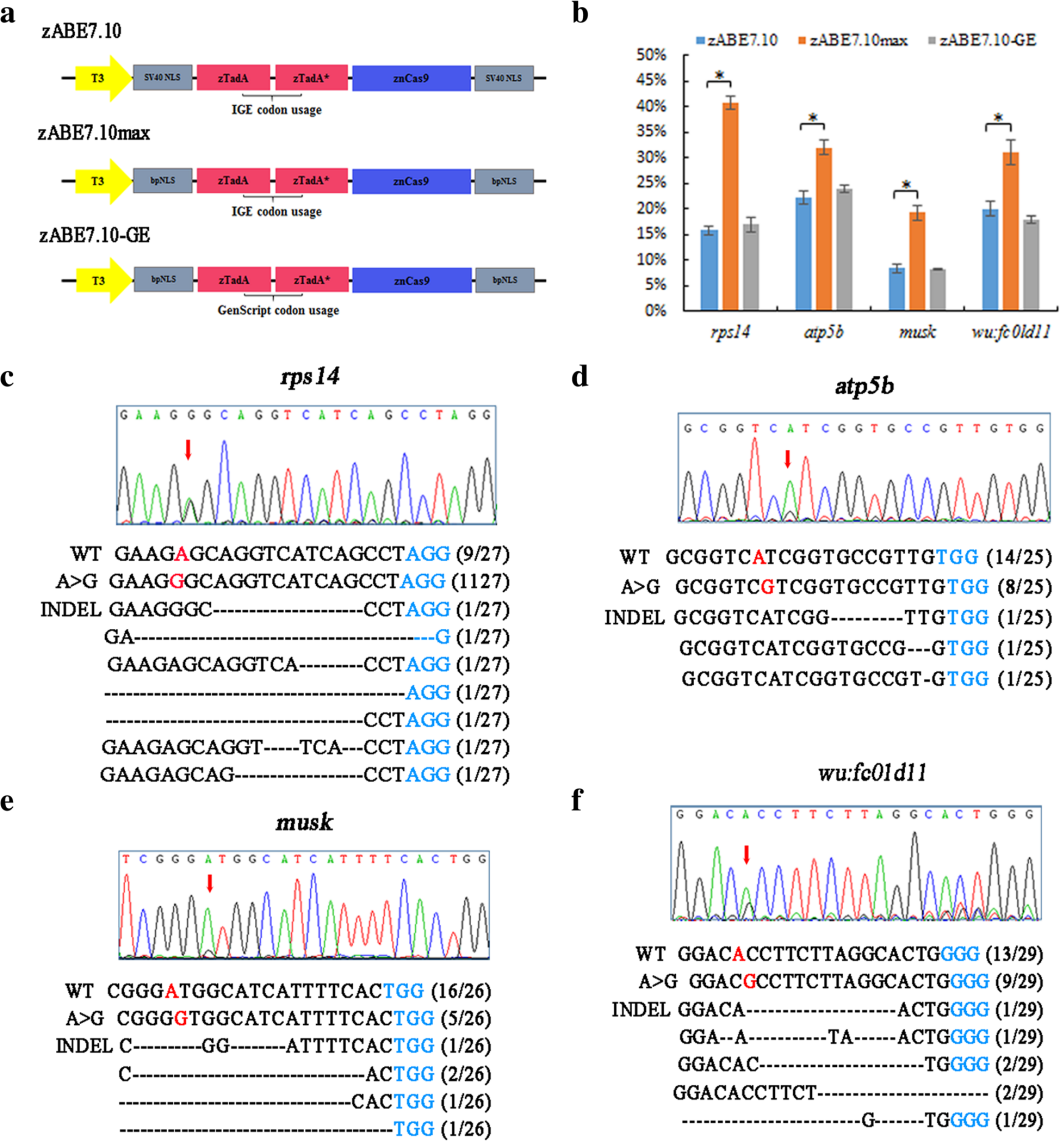
Adenine base editors modified by bpNLS enhance the base editing efficiency in zebrafish. **a** Schematic diagrams of three adenine base-editing system, zABE7.10, zABE7.10max, and zABE7.10-GE. The zABE7.10 system is a zebrafish codon-optimized version by IGE with SV40 NLS. The zABE7.10max is a version with bis-bpNLS and IGE codons. zABE7.10-GE system contain a GenScript-codon optimized sequence and bis-bpNLS. **b** The comparison of base editing efficiency of three versions of adenine base editor in zebrafish. **c**, **f** The sequencing results of the *rps14*, *atp5b*, *wu:fc01d11*, and *musk* gene in the zABE7.10max system. Target sequence (*black*), PAM region (*blue*), and the substituted bases (*red*) are indicated. The red arrows indicate the overlapped peaks. Values and error bars represent the mean of *n* = 3 biologically independent experiments. *p < 0.05

During the preparation of this manuscript, David Liu’s group reported that a new version of adenine base editor, ABEmax, modified by nuclear localization signals and codon usage, could increase the base editing efficiency in mammalian cells. So we investigated if the same strategy could be beneficial in zebrafish. We generated two zABE7.10 variants using bipartite NLS (bpNLS) and different codon usages. These two variants contained a bpNLS at both N and C termini (bis-bpNLS). The difference of the two versions is the codon usage. One is from IGE, and another is from GenScript (Fig. 4a). Next, we tested the activity of the two variants at four sites in zebrafish. Compared to zABE7.10, the new variant contained bis-bpNLS with IGE codons (zABE7.10max) had the best base editing activity at all the four sites, but the variant with GenScript (GE) codons showed no obvious improvement (Fig. 4b–f). These findings show that improvements in nuclear localization and codon usage can enhance ABE efficiency in zebrafish. Then, we tested whether the zABE7.10max could target those sites that zABE7.10 failed. Unfortunately, all the 23 sites still could not be targeted by zABE7.10max, suggesting that there are other unknown factors that affect activity of adenine base editor in zebrafish.

## Discussions and conclusions

In summary, zABE7.10 system is effective in inducing base conversion of A to G in zebrafish with high germline-targeting rate. Compared to BE system, this tool induces a little higher indels in zebrafish, which is not consistent with previous study [11]. It is worth noting that BE system could induce very high indels formation in *Xenopus laevis* [20], so there might be some animal species-specific issues associated with each gene-editing tools. Since zebrafish produce large numbers of progeny, it should be relatively easy to identify pure A → G conversion by screening more F1 embryos in zebrafish.

The original ABE7.10 editor has a base editing window from position − 17 to − 14 in protospacer in mammalian cells [11]. We found this tool had only a weak activity in zebrafish (Additional file 1: Figure S3). Codon optimization and usage of bipartite NLS greatly improved the activity of this system in zebrafish. In our study, sequencing analysis revealed that only three nucleotides at position − 16 to − 14 in protospacer could be targeted by zABE7.10, suggesting that ABE tools maybe have different target position preference in different species. Additionally, only A to G mutation can be detected using zABE7.10 system, which is similar to the finding from previous study in rice [13].

Although ABE7.9, ABE7.8, and ABE6.3 could expand the base-editing window in human cells, these modified versions did not work efficiently in zebrafish, as tested in our experiments. Recently, Kim group reported that extended gRNA could broaden ABE system’s editing window from positions − 17 to − 14 in protospacer to positions − 19 to − 14 in mouse [14]. It remains to be tested if the same strategies can work in zebrafish.

As previously shown, factors such as chromatin accessibility [21], gRNA secondary structure [22] and using Cas9 ribonucleoproteins (RNPs) [23] can influence CRISPR-Cas9 targeting activity in zebrafish. However, in our study, the A → G activity of zABE7.10 system does not have strong relationship with the specific cleavage activity of associated Cas9/gRNA. It needs to be further studied why there still are many sites that cannot be targeted by zABE7.10 in zebrafish. Although zABE7.10 is not perfect, in conclusion, it provides a new strategy for specific A → G base editing at least for some genes in zebrafish.

## Methods

### Zebrafish husbandry and maintenance

Wild-type TU zebrafish were raised and bred at 28.5 °C in a circulating system according to standard methods.

### Plasmid construction

pCMV-ABE7.10 was a gift from David Liu (Addgene plasmid #102919). The zebrafish codon-optimized wild-type ecTadA and its mutant form ecTadA*7.10 were synthesized by Guangzhou IGE Biotechnology LTD and GenScript. The synthesized products were sub-cloned into pT3TS-nCas9n vector Addgene (#46757) to generate zABE7.10 and its variants. For zABE7.9, zABE7.8, and zABE6.3 vectors, the same mutation were generated as previously described using Vazyme Mut Express II Fast Mutagenesis Kit V2 [11]. The bipartite NLS sequence was synthesized by IGE and cloned into zABE7.10 and its variants instead of SV40 T-antigen NLS using ClonExpress II One Step Cloning Kit (Vazyme).

### In vitro synthesis of capped mRNA and related gRNA

The capped mRNA was synthesized using Ambion mMESSAGE mMACHINE kit (Ambion). All the related gRNA templates were prepared according to the cloning-independent gRNA generation method [24]. The gRNAs were synthesized by Ambion MAXIscript Kit (Ambion). The capped mRNA and related gRNAs were purified using an RNeasy FFPE kit (Qiagen).

### Microinjection

A mixture (2 nL) containing zABE 7.10 mRNA (200 ng/μL) and gRNA (50 ng/μL) was coinjected into one cell-stage zebrafish embryos. Injected embryos were incubated at 28 °C for the following experiments. Injected embryos with apparently normal phenotypes were used for the following analysis.

### Evaluation of base editing efficiency

After 2dpf, genomic DNA from individual embryos or pools of six injected embryos that developed normally was extracted by alkaline lysis buffer-based DNA extraction. The targeted genomic loci were amplified from genomic DNA and the PCR products were used for both direct sequencing and T-A cloning/sequencing. For PCR direct products sequencing, we evaluated three groups (6 embryos in one group). For T-A cloning/sequencing, 30 to 36 clones were chosen to sequence. As for off-target analysis, we sequenced 30 colonies for each site. All the used primers are listed in Additional file 1: Table S3. T7EI assays were performed as previously described [25]. The efficiency of the CRISPR/Cas9 system was confirmed by electrophoresis on a 2% agarose gel (Additional file 1: Figure S4). Quantification was based on relative band intensity using Quantity One software (Bio-Rad). For calculating the germline targeting efficiency and the germline transmission rate, injected embryos were raised to adults of F0 and matted to wild-type fish to produce F1 fish. For germline-targeting efficiency, Founders could be identified from sequencing results of three pools of six F1 embryos by observing the overlapped peaks at the targeted site. For germline transmission rate, we randomly selected 24 F1 embryos and sequenced individually.

### Imaging

Zebrafish embryos were anesthetized with 0.03% Tricaine (Sigma-Aldrich) and mounted in 4% methylcellulose. Photographs were taken by a Zeiss Axio Imager Z1 microscope and processed by Adobe Photoshop CC software.

### *o*-dianisidine staining and genotyping

Embryos were first anesthetized with Tricane (Sigma-Aldrich) and then fixed at room temperature for 2 h using 4% paraformaldehyde (PFA) in PBS. To remove PFA, three washes (5 min each) with PBS were performed. Next, embryos were stained with 0.6 mg/mL o-dianisidine (Sigma, USA) in an o-dianisidine staining solution (40% ethanol, 0.65% H_2_O_2_, 10 mmol/L Na-Acetate) for 30 min in dark and followed by four wash times (5 min each) with PBS. Then, embryos were incubated into a bleach solution (1% KOH, 3% H_2_O_2_) for 20 min to remove pigmentation. Finally, the embryos were washed with PBS and imaged immediately. All the embryos with a defect of erythroid cell were selected by imaging and ultimately its genotype confirmed by sequencing.

### Statistics and reproducibility

Experiments were repeated for three times independently. Then, the mean and SEM were calculated. *P* values were calculated using a two-sided unpaired Student’s *t* test and less than 0.05 was considered as significant.

## Additional file

Additional file 1 Figure S1. The optimized zABE7.10 system induce indels formation during base editing. Figure S2. Base-editing frequency at potential off-targets in zABE7.10 system of zebrafish genome. Figure S3. Low efficiency of base conversion induced by the original ABE7.10 editor in zebrafish at *rps14* locus. Figure S4. Target efficiency measured by T7EI assays. Table S1. The Cas9 cleavage activity of 23 target sites used in this study. Table S2. Germline transmission rate of the *ddx17, rps14, atp5b, wu:fc01d11* and *musk* gene. Table S3**.** Primers used in this study. (DOCX 918 kb)
