# Supplementary material for: Precise A•T to G•C base editing in the zebrafish genome
Source: BMC Biol. 2018 Nov 20;16:139. doi: 10.1186/s12915-018-0609-1 (PMC6247682; doi:10.1186/s12915-018-0609-1)
Supplement: Supplementary file 1 — Figure S1. The optimized zABE7.10 system induce indels formation during base editing. Figure S2. Base-editing frequency at potential off-targets in zABE7.10 system of zebrafish genome. Figure S3. Low efficiency of base conversion induced by the original ABE7.10 editor in zebrafish at rps14 locus. Figure S4. Target efficiency measured by T7EI assays. Table S1. The Cas9 cleavage activity of 23 target sites used in this study. Table S2. Germline transmission rate of the ddx17, rps14, atp5b, wu:fc01d11 and musk gene. Table S3. Primers used in this study. (DOCX 918 kb) [file 12915_2018_609_MOESM1_ESM.docx]

Additional file 1

Precise A.T to G.C base editing in the zebrafish genome

Wei Qin^1, †,^ *, Xiaochan Lu^1, †^, Yunxing Liu^1^, Haipeng Bai^1^, Song Li^1^ and Shuo Lin^1, 2,^*

**Supplementary Figures**

**
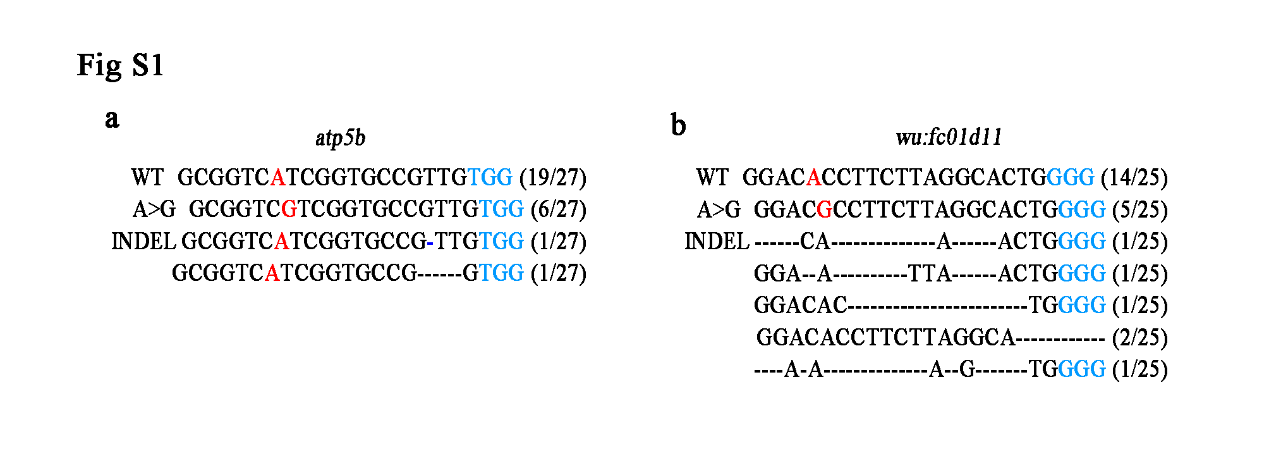
**

**Figure S1. The optimized zABE7.10 system induce indels formation during base editing. a,b.** The sequencing results of T-A cloning show that the indels formation could detected both in *atp5b* and *wu:fc01d11*. Target sequence (*black*), PAM region (*blue*) and the substituted bases (*red*) are indicated. The black dashes and blue dashes denote deleted bases and inserted bases in the sequence, respectively.


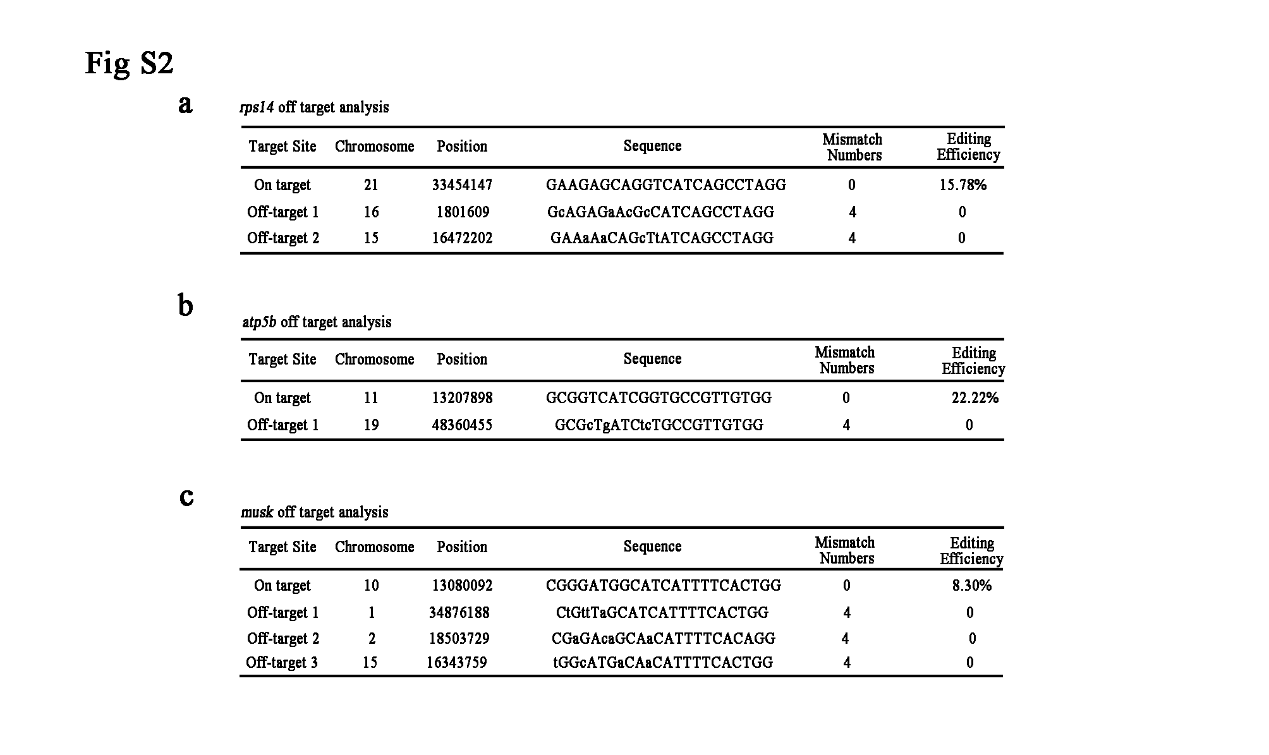


**Figure S2. Base editing frequency at potential off-targets in zABE7.10 system of zebrafish genome. a,b,c.** Substitution frequencies at potential off-target sites of *rps14*, *atp5b* and *musk* were shown. The mismatch bases in potential off-target sites are indicated in lowercase.


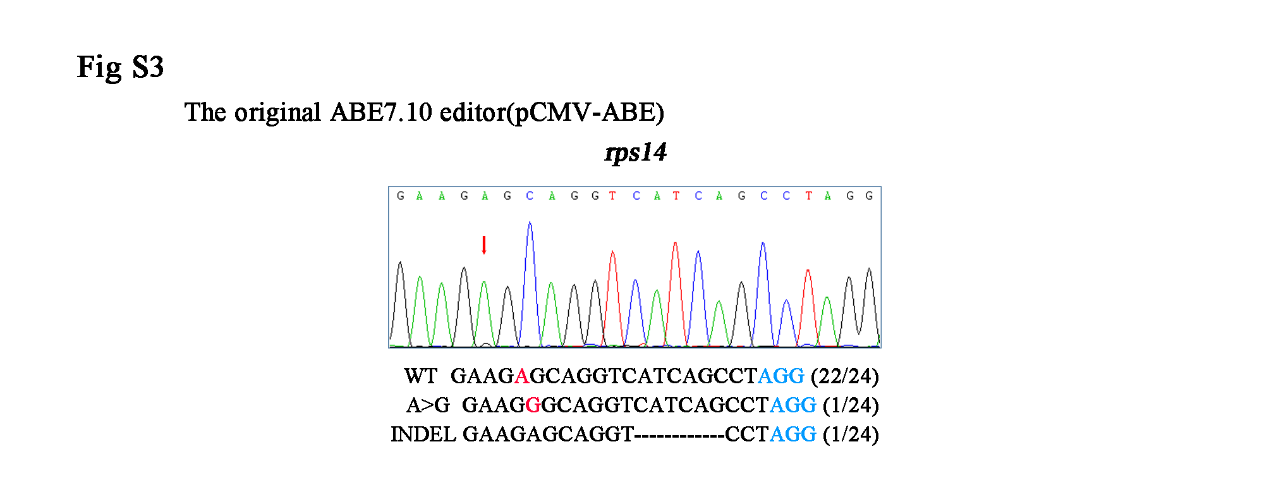


**Figure S3. Low efficiency of base conversion induced by the original ABE7.10 editor in zebrafish at *rps14* locus.** Sequence chromatograms of *rps14* locus was shown. The red arrows indicate the overlapped peaks.


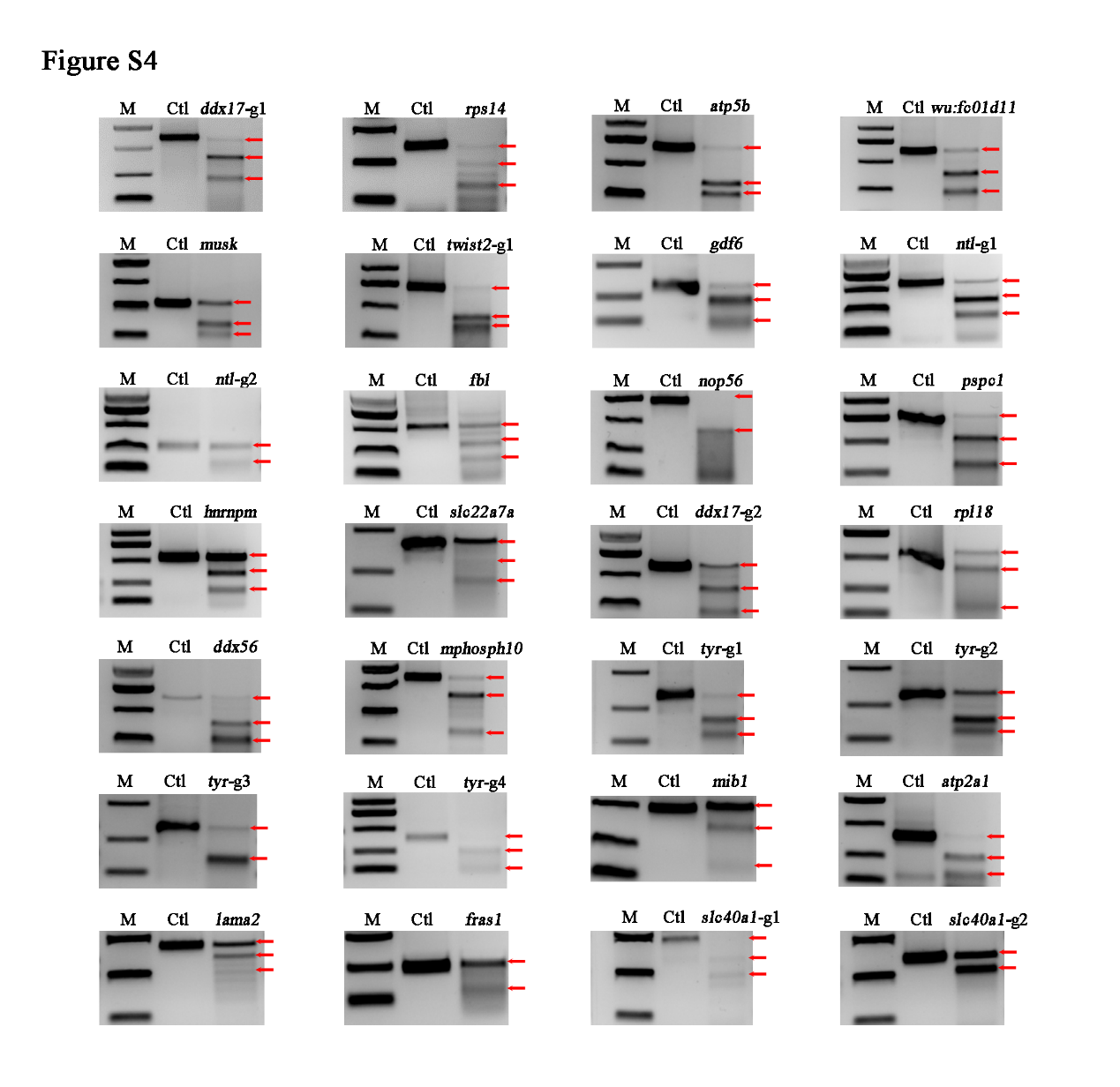


**Figure S4. Target efficiency measured by T7EI assays.** The T7EI results of 28 targets sites in CRISPR/Cas9 system.

**Supplementary Table**

**Table S1. The Cas9 cleavage activity of 23 target sites used in this study.**

| **Gene** | **Target site** | **Cas9 Cleavage Activity** |
| --- | --- | --- |
| *twist2 g1* | GCTCCAGAACCAGCGCGTCCTGG | 81.83% |
| *gdf6* | GTCGATCAGAGAGGCCACTGG | 42.56% |
| *ntl-g1* | GGAACCAGCCACCGACTGTTGG | 52.48% |
| *ntl-g2* | TTCAGAAGGGCAGCGAGAAAGGG | 15.52% |
| *fbl* | GGTGGAGGATTCAAGTCTCCGG | 27.79% |
| *nop56* | GTGGTAGCTGAAATCATAAGAGG | 100% |
| *pspc1* | GATGATCGAGGCCGGCCCACAGG | 68.56% |
| *hnrnpm* | GTCCAACCATGAATCGAGAAAGG | 36.48% |
| *slc22a7a* | GGCATCCCACGTAATTCAGATGG | 11.23% |
| *ddx17-g2* | GACTCATGTGATGTACTTCTGGG | 39.78% |
| *rpl18* | GGGAGAGAGCCAGAGGTGGGCGG | 36.70% |
| *ddx56* | GTGAGGACTCTCTGGATCAGTGG | 74.50% |
| *mphosph10* | GAGAGGAGGAAACTACACAGAGG | 68.15% |
| *tyr g1* | GGCTGACGGACTACGAGACCGGG | 79.67% |
| *tyr g2* | CCTGAGCTTCAGGAACGCGCTGG | 50.26% |
| *tyr g3* | TGAAGCTCAGGTTTGCCCGTCGG | 58.80% |
| *tyr g4* | GCTCCAGCATCTTTGAGCAGTGG | 62.06% |
| *mib1* | GGTCAACATTGATTTGGACCTGG | 23.31% |
| *atp2a1* | TTCAACGACCCCGTCCATGGTGG | 76.64% |
| *lama2* | CAGGATTTCTGAGATCACTCTGG | 38.73% |
| *fras1* | CATGACAAGCTCTTGTTTTTAGG | 19.93% |
| *slc40a1-g1* | CCCAGACTAAAAGGTAAACAAGG | 100% |
| *slc40a1-g2* | AAGAGACTGGGTTGTGGTTGTGG | 43.58% |

**Table S2. Germline transmission rate of the *ddx17*-g1*, rps14, atp5b, wu:fc01d11* and *musk* gene**

| **Gene** | **Cross** | **Indels transmission rate** | **Targeted adenine transmission rate** |
| --- | --- | --- | --- |
| *ddx17*-g1 | #1 | 8.33%(2/24) | 41.67%(10/24) |
| *rps14* | #3 | 12.5%(3/24) | 25%(6/24) |
| *atp5b* | #4 | 16.67%(4/24) | 58.33%(14/24) |
| *wu:fc01d11* | #2 | 29.16%(7/24) | 37.5%(9/24) |
| *musk* | #3 | 12.5%(3/24) | 45.83%(11/24) |

**Table S3. Primers used in this study.**

| **Gene name** | **Sequence of the primer (5’-3’)** |
| --- | --- |
| *ddx17* | F: TGGAACTTGTTTGTCCATGTGT |
|  | R: ACGCCAGCTTCCATTAACCA |
| *rps14* | F: CGTGATGTATGTCGTTATCTAGAGATG |
|  | R: GCATGTGCACACAAAGACAC |
| *atp5b* | F: CTGCGACTTCGTGTCATTGTG |
|  | R: CCACAGGGATTCTGATGGGG |
| *wu:fc01d11* | F: AACAGCTGCTGAAAGAAGAACC |
|  | R: TGAAGCTTTTGATGCACATTCT |
| *musk* | F: GGAATGGCGTACTTATCGGAGC |
|  | R: GAAGATTTCCCATAATACCAC |
| *twist2* | F: AAGTGGAGATCGTATTTTCTC |
|  | R: GTTCGGCATTATCAGGACGCG |
| *gdf6* | F: GCTCTCTCCTTTGCGAAGAC |
|  | R: AATCCCCCAAACGTCCAGAA |
| *ntl* g1 | F: GCCAAAGCTTTCCTCGATGC |
|  | R: TTGCTCTTACTGGTGGTAGTGC |
| *ntl* g2 | F: CGGGATTTAGTAGGATCGTC |
|  | R: TGACAATCATTTCATTGGTG |
| *fbl* | F: ATAGATGGATGGATGGATGG |
|  | R: CCTCCTTACCACGGCAGATA |
| *nop56* | F: GATGCACACATAATCTGTCAA |
|  | R: CTAGAGAGCATTTGATTGGT |
| *pspc1* | F: GGCCAGTGTTTCAGTTTAATG |
|  | R: GCCCATCTTCATCATCATAT |
| *hnrnpm* | F: CTGCAATCAACTCAAACCTG |
|  | R: CGAGACCACTTCCCCATGAT |
| *slc22a7a* | F: CCGCGGGTCATTCTTCCTT |
|  | R: CCTTACCTCAGTGGCAGTTGT |
| *rpl18* | F: ATGCGTCAGTTTTGGCCAGT |
|  | R: TGTGGACGGAGAGCTTTTCA |
| *ddx56* | F: ACATTGACTTTCTGTTAAGGCACA |
|  | R: CCGTTTCAACATTAAGTTGGGATT |
| *mphosph10* | F: AGTGTCCAGGATGTTTTGGCA |
|  | R: CAGACTGAGTCCAGTTCGGT |
| *tyr* g1-3 | F: TGCTAATGTCGTTCACTCTG |
|  | R: CCGTTCATTCATGTCAAGGA |
| *tyr* g4 | F: TCATCAAATCAAATCAGCTG |
|  | R: GAGTGTGTGTGTTGGCTAAT |
| *mib1* | F: AGCCTTAGATGATAATGATC |
|  | R: ACAAACTACATTGGCACTGC |
| *atp2a1* | F: GGCTATTGGAGTTGTGGTTG |
|  | R: CCATTGATGCTTGATATTTAGG |
| *lama2* | F: GCTCCATGATAACGGCCTATG |
|  | R: GATGCAGATCCACCAGCCAG |
| *fras1* | F: CATGCTCAGCTGGTTGTGTG |
|  | R: TGATGAGAACAGGCCTTAATGA |
| *slc40a1*-g1 | F: CTGGACTGCTTCATTGTGTC |
|  | R: CCAAAGATGTCTGTGCCACTG |
| *slc40a1*-g2 | F: GCAGACAAAATGAGGCTCGCTG |
|  | R: AGCCGCTGTAATGGAATCTC |
| *rps14* off-target 1 | F: GGACGGGCTAATAATTCAAC |
|  | R: TGAAGCCTGACTTTGACATC |
| *rps14* off-target 2 | F: AAGGACATGGGTAGTTGACG |
|  | R: ACGTCCAGCTTAAACCAGGC |
| *atp5b* off-target 1 | F: CGTCCGTGTCCAGCTCTCTC |
|  | R: AGCTGCACACCATACCTCAG |
| *musk* off-target 1 | F: GATTGCACTACCAAATTAC |
|  | R: ATCCTCACCAATAAGCTGAC |
| *musk* off-target 2 | F: TAGCATGTCTCCAGTGTTCC |
|  | R: CTACATAATAGCATGCTCAG |
| *musk* off-target 3 | F: GAAATGAGTGGGATGCTTCG |
|  | R: CAATTTGTCTAGACTACCAC |

**Supplementary Sequences**

**The sequence of the zABE7.10, zABE7.10max and zABE7.10-GE in this study. Color coding is as follows:**

**Green = SV40 NLS or bipartite NLS**

**Purple = Zebrafish codon optimized Cas9 sequence**

Orange **= Linker**

**Blue = Zebrafish codon optimized ecTadA and ecTadA*7.10 sequence (IGE or GenScript)**

**Red = Start codon and Stop codon**

**zABE7.10**

ATGGCTTCTCCACCTAAGAAGAAGAGAAAGGTGGGAAGCATGAGCGAAGTCGAGTTTTCACACGAATACTGGATGAGACATGCTCTCACACTCGCAAAGAGGGCTTGGGACGAGCGCGAGGTTCCTGTTGGCGCCGTGCTGGTTCATAATAACAGAGTGATCGGAGAAGGGTGGAACAGACCTATTGGGAGACATGATCCAACCGCCCATGCCGAGATCATGGCTCTGCGCCAGGGCGGACTGGTGATGCAGAACTACAGACTGATAGATGCCACTCTGTATGTGACTCTGGAGCCTTGCGTGATGTGCGCTGGAGCTATGATTCACTCTCGCATTGGACGCGTGGTCTTTGGTGCACGCGATGCAAAGACCGGCGCTGCAGGATCTCTGATGGACGTCCTGCACCACCCAGGCATGAACCACAGAGTGGAGATAACTGAGGGTATCCTGGCAGACGAGTGTGCCGCCCTGCTGTCTGATTTCTTTAGAATGCGCCGCCAGGAAATTAAGGCCCAGAAGAAAGCCCAATCAAGTACAGACAGCGGAGGTTCCAGCGGAGGCTCCAGTGGAAGCGAAACACCTGGGACATCAGAGTCTGCCACTCCAGAGTCCAGTGGAGGCTCCTCTGGCGGCAGTAGCGAGGTCGAATTTTCCCATGAATATTGGATGAGGCACGCTCTGACCCTCGCCAAAAGAGCTAGAGATGAGAGGGAGGTTCCTGTGGGCGCAGTGCTGGTCCTGAATAACAGGGTTATAGGCGAGGGATGGAACCGCGCAATTGGACTGCATGACCCAACCGCACACGCAGAGATTATGGCACTCAGGCAAGGAGGACTGGTCATGCAGAACTATAGACTCATTGACGCCACACTGTATGTCACCTTCGAGCCTTGTGTGATGTGCGCAGGAGCCATGATCCACAGTAGGATCGGTAGAGTCGTGTTTGGAGTTAGAAACGCAAAGACCGGAGCTGCCGGCAGTCTGATGGATGTTCTGCACTACCCAGGAATGAATCATAGGGTCGAGATCACCGAAGGCATTCTGGCCGACGAATGTGCCGCTCTGCTGTGCTATTTCTTCAGAATGCCAAGACAGGTTTTCAACGCTCAGAAGAAAGCCCAGTCTTCCACTGATAGCGGCGGTAGTTCTGGAGGGTCCTCAGGCTCAGAAACTCCTGGAACTTCCGAGAGCGCCACTCCCGAATCATCAGGCGGCAGTAGCGGAGGATCT**GATAAGAAGTATAGCATCGGCCTGGCTATTGGAACTAACTCCGTGGGTTGGGCAGTGATTACAGACGACTACAAGGTCCCTAGCAAGAAATTTAAGGTGCTGGGTAACACCGACAGGCACAGCATCAAGAAAAATCTGATTGGAGCCCTGCTGTTCGGTTCTGGAGAGACTGCCGAAGCAACACGCCTGAAAAGAACAGCAAGAAGGCGCTATACCAGAAGGAAGAATAGAATCTGTTACCTGCAGGAGATTTTCTCTAACGAAATGGCTAAGGTGGACGATTCATTCTTTCATAGGCTGGAGGAAAGTTTCCTGGTCGAGGAAGATAAGAAACACGAGCGCCATCCTATCTTTGGAAACATTGTGGACGAGGTCGCCTATCACGAAAAATACCCAACCATCTATCATCTGCGCAAGAAACTGGCTGACTCTACTGATAAAGCCGACCTGAGACTGATCTATCTGGCTCTGGCCCACATGATTAAGTTCAGGGGTCATTTTCTGATCGAGGGCGATCTGAACCCCGACAATTCCGATGTGGACAAGCTGTTCATCCAGCTGGTCCAGATTTACAATCAGCTGTTTGAGGAAAACCCTATTAATGCTTCCAGAGTGGACGCAAAAGCTATCCTGTCAGCCAGGCTGTCCAAGTCACGCAGACTGGAGAACCTGATTGCACAGCTGCCCGGAGAAAAGAGGAACGGTCTGTTTGGAAATCTGATCGCTCTGAGTCTGGGCCTGACTCCTAACTTCAAAAGCAATTTTGATCTGGCTGAGGACGCCAAACTGCAGCTGTCAAAGGACACATATGACGATGACCTGGATAACCTGCTGGCACAGATCGGAGATCAGTACGCTGACCTGTTCCTGGCTGCCAAAAATCTGTCCGACGCAATCCTGCTGTCAGATATTCTGAGAGTGAACAGCGAGATTACAAAAGCTCCTCTGAGTGCCAGCATGATCAAGAGATATGACGAGCACCATCAGGATCTGACCCTGCTGAAGGCTCTGGTCAGGCAGCAGCTGCCAGAGAAGTACAAGGAAATTTTCTTTGATCAGTCCAAGAACGGCTACGCCGGTTATATCGACGGAGGCGCATCACAGGAGGAATTCTACAAGTTTATCAAACCTATTCTGGAGAAGATGGACGGAACTGAGGAACTGCTGGTGAAACTGAATAGAGAGGACCTGCTGAGGAAGCAGCGCACATTTGATAACGGTTCCATCCCACACCAGATTCATCTGGGAGAGCTGCACGCTATCCTGAGGCGCCAGGAAGACTTCTACCCCTTTCTGAAAGATAACCGCGAGAAGATCGAAAAAATTCTGACCTTCAGAATCCCTTACTATGTGGGTCCACTGGCTCGCGGAAACAGCAGATTTGCCTGGATGACTCGCAAATCCGAGGAAACCATTACTCCTTGGAACTTCGAGGAAGTGGTCGATAAGGGCGCCTCTGCACAGTCCTTCATCGAGAGAATGACTAATTTTGACAAAAACCTGCCCAATGAGAAAGTGCTGCCTAAGCACTCCCTGCTGTACGAGTATTTCACTGTCTATAACGAACTGACAAAGGTGAAATACGTCACCGAGGGCATGAGAAAGCCAGCCTTCCTGTCAGGAGAGCAGAAGAAAGCAATCGTGGATCTGCTGTTTAAAACCAACAGGAAAGTGACTGTCAAGCAGCTGAAGGAGGACTACTTCAAGAAAATTGAATGCTTCGATTCCGTGGAGATCAGCGGAGTCGAAGACAGATTTAACGCAAGCCTGGGCGCTTACCACGATCTGCTGAAGATCATTAAGGATAAAGACTTCCTGGACAACGAGGAAAATGAGGATATCCTGGAAGACATTGTGCTGACACTGACCCTGTTTGAGGACAGAGGAATGATCGAGGAAAGACTGAAAACCTATGCTCATCTGTTCGATGACAAGGTGATGAAACAGCTGAAGAGAAGGCGCTACACTGGCTGGGGTAGACTGAGCAGGAAGCTGATCAACGGCATTAGGGATAAACAGTCAGGAAAGACAATCCTGGACTTTCTGAAAAGTGATGGCTTCGCCAACCGCAATTTTATGCAGCTGATTCACGATGACAGTCTGACCTTCAAAGAGGACATCCAGAAGGCTCAGGTGTCTGGACAGGGCCACTCCCTGCATGAGCAGATTGCAAACCTGGCTGGAAGCCCAGCCATCAAGAAAGGCATTCTGCAGACAGTGAAAATCGTCGATGAGCTGGTGAAAGTCATGGGCCATAAGCCCGAAAACATCGTGATTGAGATGGCTCGCGAAAATCAGACAACCCAGAAGGGTCAGAAGAACAGTAGAGAGAGGATGAAAAGAATCGAGGAAGGCATTAAGGAGCTGGGTAGCCAGATCCTGAAAGAGCACCCAGTGGAAAACACACAGCTGCAGAATGAGAAGCTGTATCTGTACTATCTGCAGAATGGAAGAGATATGTACGTGGACCAGGAGCTGGATATTAACAGGCTGTCTGATTACGACGTGGATCATATCGTCCCCCAGAGTTTCATCAAAGATGACAGCATTGACAACAAGGTGCTGACCAGGTCCGACAAAAACAGAGGAAAATCAGATAATGTCCCTAGTGAGGAAGTGGTCAAGAAAATGAAGAACTACTGGAGACAGCTGCTGAATGCCAAACTGATCACTCAGAGGAAGTTTGATAACCTGACAAAAGCAGAGCGCGGTGGACTGTCAGAACTGGACAAAGCTGGATTCATCAAGAGGCAGCTGGTGGAAACACGCCAGATCACTAAACACGTCGCACAGATTCTGGATAGTCGCATGAACACAAAGTACGATGAGAATGACAAACTGATCAGAGAAGTGAAGGTCATTACCCTGAAGAGTAAACTGGTCAGCGACTTTAGGAAAGATTTCCAGTTTTATAAGGTCCGCGAGATTAACAATTATCACCATGCCCATGACGCATACCTGAACGCCGTGGTCGGTACCGCACTGATCAAGAAATACCCAAAACTGGAGAGCGAATTCGTGTACGGAGACTATAAGGTGTACGATGTCAGAAAAATGATCGCCAAGTCCGAGCAGGAAATTGGAAAAGCTACTGCCAAGTATTTCTTTTACTCAAACATCATGAATTTCTTTAAGACAGAGATCACCCTGGCCAATGGAGAAATCCGCAAAgcGCCCCTGATTGAGACAAACGGAGAGACAGGCGAAATCGTGTGGGACAAAGGCAGAGATTTTGCAACCGTGAGGAAGGTCCTGAGCATGCCTCAAGTGAATATCGTCAAGAAAACTGAGGTGCAGACAGGCGGTTTCTCAAAAGAAAGTATTCTGCCAAAACGCAACTCTGATAAGCTGATCGCTAGAAAGAAAGACTGGGACCCTAAGAAGTATGGAGGCTTTGACTCTCCCACTGTGGCATACTCCGTCCTGGTGGTCGCTAAGGTGGAGAAGGGCAAAAGCAAGAAACTGAAATCTGTCAAGGAGCTGCTGGGTATCACAATTATGGAGAGAAGCTCTTTCGAGAAGAACCCAATCGATTTTCTGGAGGCCAAAGGTTATAAGGAAGTGAAGAAAGACCTGATCATTAAACTGCCCAAGTACAGTCTGTTTGAGCTGGAAAACGGCAGGAAACGCATGCTGGCAAGCGCTGGAGAGCTGCAGAAAGGCAATGAACTGGCCCTGCCTTCTAAGTACGTGAACTTCCTGTATCTGGCAAGCCACTACGAGAAGCTGAAAGGATCTCCAGAGGATAACGAACAGAAACAGCTGTTTGTGGAGCAGCACAAGCATTATCTGGACGAGATCATTGAACAGATTAGCGAGTTCTCTAAAAGAGTGATCCTGGCCGACGCAAATCTGGATAAGGTCCTGTCTGCTTACAACAAACACAGAGATAAGCCCATCAGGGAGCAGGCCGAAAATATCATTCATCTGTTCACTCTGACAAACCTGGGCGCACCTGCAGCTTTCAAGTACTTCGACACTACAATCGATAGAAAGAGGTACACCTCCACTAAGGAGGTGCTGGACGCTACACTGATCCATCAGAGTATTACCGGCCTGTACGAAACAAGGATTGACCTGTCTCAGCTGGGTGGCGACTcaccggtgagatctcctaagaagaagagaaaggtgTGA**

**aABE7.10max**

ATGAAAAGAACTGCAGATGGATCCGAGTTCGAGAGCCCCAAGAAAAAGAGAAAGGTGGGAAGCATGAGCGAAGTCGAGTTTTCACACGAATACTGGATGAGACATGCTCTCACACTCGCAAAGAGGGCTTGGGACGAGCGCGAGGTTCCTGTTGGCGCCGTGCTGGTTCATAATAACAGAGTGATCGGAGAAGGGTGGAACAGACCTATTGGGAGACATGATCCAACCGCCCATGCCGAGATCATGGCTCTGCGCCAGGGCGGACTGGTGATGCAGAACTACAGACTGATAGATGCCACTCTGTATGTGACTCTGGAGCCTTGCGTGATGTGCGCTGGAGCTATGATTCACTCTCGCATTGGACGCGTGGTCTTTGGTGCACGCGATGCAAAGACCGGCGCTGCAGGATCTCTGATGGACGTCCTGCACCACCCAGGCATGAACCACAGAGTGGAGATAACTGAGGGTATCCTGGCAGACGAGTGTGCCGCCCTGCTGTCTGATTTCTTTAGAATGCGCCGCCAGGAAATTAAGGCCCAGAAGAAAGCCCAATCAAGTACAGACAGCGGAGGTTCCAGCGGAGGCTCCAGTGGAAGCGAAACACCTGGGACATCAGAGTCTGCCACTCCAGAGTCCAGTGGAGGCTCCTCTGGCGGCAGTAGCGAGGTCGAATTTTCCCATGAATATTGGATGAGGCACGCTCTGACCCTCGCCAAAAGAGCTAGAGATGAGAGGGAGGTTCCTGTGGGCGCAGTGCTGGTCCTGAATAACAGGGTTATAGGCGAGGGATGGAACCGCGCAATTGGACTGCATGACCCAACCGCACACGCAGAGATTATGGCACTCAGGCAAGGAGGACTGGTCATGCAGAACTATAGACTCATTGACGCCACACTGTATGTCACCTTCGAGCCTTGTGTGATGTGCGCAGGAGCCATGATCCACAGTAGGATCGGTAGAGTCGTGTTTGGAGTTAGAAACGCAAAGACCGGAGCTGCCGGCAGTCTGATGGATGTTCTGCACTACCCAGGAATGAATCATAGGGTCGAGATCACCGAAGGCATTCTGGCCGACGAATGTGCCGCTCTGCTGTGCTATTTCTTCAGAATGCCAAGACAGGTTTTCAACGCTCAGAAGAAAGCCCAGTCTTCCACTGATAGCGGCGGTAGTTCTGGAGGGTCCTCAGGCTCAGAAACTCCTGGAACTTCCGAGAGCGCCACTCCCGAATCATCAGGCGGCAGTAGCGGAGGATCT**GATAAGAAGTATAGCATCGGCCTGGCTATTGGAACTAACTCCGTGGGTTGGGCAGTGATTACAGACGACTACAAGGTCCCTAGCAAGAAATTTAAGGTGCTGGGTAACACCGACAGGCACAGCATCAAGAAAAATCTGATTGGAGCCCTGCTGTTCGGTTCTGGAGAGACTGCCGAAGCAACACGCCTGAAAAGAACAGCAAGAAGGCGCTATACCAGAAGGAAGAATAGAATCTGTTACCTGCAGGAGATTTTCTCTAACGAAATGGCTAAGGTGGACGATTCATTCTTTCATAGGCTGGAGGAAAGTTTCCTGGTCGAGGAAGATAAGAAACACGAGCGCCATCCTATCTTTGGAAACATTGTGGACGAGGTCGCCTATCACGAAAAATACCCAACCATCTATCATCTGCGCAAGAAACTGGCTGACTCTACTGATAAAGCCGACCTGAGACTGATCTATCTGGCTCTGGCCCACATGATTAAGTTCAGGGGTCATTTTCTGATCGAGGGCGATCTGAACCCCGACAATTCCGATGTGGACAAGCTGTTCATCCAGCTGGTCCAGATTTACAATCAGCTGTTTGAGGAAAACCCTATTAATGCTTCCAGAGTGGACGCAAAAGCTATCCTGTCAGCCAGGCTGTCCAAGTCACGCAGACTGGAGAACCTGATTGCACAGCTGCCCGGAGAAAAGAGGAACGGTCTGTTTGGAAATCTGATCGCTCTGAGTCTGGGCCTGACTCCTAACTTCAAAAGCAATTTTGATCTGGCTGAGGACGCCAAACTGCAGCTGTCAAAGGACACATATGACGATGACCTGGATAACCTGCTGGCACAGATCGGAGATCAGTACGCTGACCTGTTCCTGGCTGCCAAAAATCTGTCCGACGCAATCCTGCTGTCAGATATTCTGAGAGTGAACAGCGAGATTACAAAAGCTCCTCTGAGTGCCAGCATGATCAAGAGATATGACGAGCACCATCAGGATCTGACCCTGCTGAAGGCTCTGGTCAGGCAGCAGCTGCCAGAGAAGTACAAGGAAATTTTCTTTGATCAGTCCAAGAACGGCTACGCCGGTTATATCGACGGAGGCGCATCACAGGAGGAATTCTACAAGTTTATCAAACCTATTCTGGAGAAGATGGACGGAACTGAGGAACTGCTGGTGAAACTGAATAGAGAGGACCTGCTGAGGAAGCAGCGCACATTTGATAACGGTTCCATCCCACACCAGATTCATCTGGGAGAGCTGCACGCTATCCTGAGGCGCCAGGAAGACTTCTACCCCTTTCTGAAAGATAACCGCGAGAAGATCGAAAAAATTCTGACCTTCAGAATCCCTTACTATGTGGGTCCACTGGCTCGCGGAAACAGCAGATTTGCCTGGATGACTCGCAAATCCGAGGAAACCATTACTCCTTGGAACTTCGAGGAAGTGGTCGATAAGGGCGCCTCTGCACAGTCCTTCATCGAGAGAATGACTAATTTTGACAAAAACCTGCCCAATGAGAAAGTGCTGCCTAAGCACTCCCTGCTGTACGAGTATTTCACTGTCTATAACGAACTGACAAAGGTGAAATACGTCACCGAGGGCATGAGAAAGCCAGCCTTCCTGTCAGGAGAGCAGAAGAAAGCAATCGTGGATCTGCTGTTTAAAACCAACAGGAAAGTGACTGTCAAGCAGCTGAAGGAGGACTACTTCAAGAAAATTGAATGCTTCGATTCCGTGGAGATCAGCGGAGTCGAAGACAGATTTAACGCAAGCCTGGGCGCTTACCACGATCTGCTGAAGATCATTAAGGATAAAGACTTCCTGGACAACGAGGAAAATGAGGATATCCTGGAAGACATTGTGCTGACACTGACCCTGTTTGAGGACAGAGGAATGATCGAGGAAAGACTGAAAACCTATGCTCATCTGTTCGATGACAAGGTGATGAAACAGCTGAAGAGAAGGCGCTACACTGGCTGGGGTAGACTGAGCAGGAAGCTGATCAACGGCATTAGGGATAAACAGTCAGGAAAGACAATCCTGGACTTTCTGAAAAGTGATGGCTTCGCCAACCGCAATTTTATGCAGCTGATTCACGATGACAGTCTGACCTTCAAAGAGGACATCCAGAAGGCTCAGGTGTCTGGACAGGGCCACTCCCTGCATGAGCAGATTGCAAACCTGGCTGGAAGCCCAGCCATCAAGAAAGGCATTCTGCAGACAGTGAAAATCGTCGATGAGCTGGTGAAAGTCATGGGCCATAAGCCCGAAAACATCGTGATTGAGATGGCTCGCGAAAATCAGACAACCCAGAAGGGTCAGAAGAACAGTAGAGAGAGGATGAAAAGAATCGAGGAAGGCATTAAGGAGCTGGGTAGCCAGATCCTGAAAGAGCACCCAGTGGAAAACACACAGCTGCAGAATGAGAAGCTGTATCTGTACTATCTGCAGAATGGAAGAGATATGTACGTGGACCAGGAGCTGGATATTAACAGGCTGTCTGATTACGACGTGGATCATATCGTCCCCCAGAGTTTCATCAAAGATGACAGCATTGACAACAAGGTGCTGACCAGGTCCGACAAAAACAGAGGAAAATCAGATAATGTCCCTAGTGAGGAAGTGGTCAAGAAAATGAAGAACTACTGGAGACAGCTGCTGAATGCCAAACTGATCACTCAGAGGAAGTTTGATAACCTGACAAAAGCAGAGCGCGGTGGACTGTCAGAACTGGACAAAGCTGGATTCATCAAGAGGCAGCTGGTGGAAACACGCCAGATCACTAAACACGTCGCACAGATTCTGGATAGTCGCATGAACACAAAGTACGATGAGAATGACAAACTGATCAGAGAAGTGAAGGTCATTACCCTGAAGAGTAAACTGGTCAGCGACTTTAGGAAAGATTTCCAGTTTTATAAGGTCCGCGAGATTAACAATTATCACCATGCCCATGACGCATACCTGAACGCCGTGGTCGGTACCGCACTGATCAAGAAATACCCAAAACTGGAGAGCGAATTCGTGTACGGAGACTATAAGGTGTACGATGTCAGAAAAATGATCGCCAAGTCCGAGCAGGAAATTGGAAAAGCTACTGCCAAGTATTTCTTTTACTCAAACATCATGAATTTCTTTAAGACAGAGATCACCCTGGCCAATGGAGAAATCCGCAAAgcGCCCCTGATTGAGACAAACGGAGAGACAGGCGAAATCGTGTGGGACAAAGGCAGAGATTTTGCAACCGTGAGGAAGGTCCTGAGCATGCCTCAAGTGAATATCGTCAAGAAAACTGAGGTGCAGACAGGCGGTTTCTCAAAAGAAAGTATTCTGCCAAAACGCAACTCTGATAAGCTGATCGCTAGAAAGAAAGACTGGGACCCTAAGAAGTATGGAGGCTTTGACTCTCCCACTGTGGCATACTCCGTCCTGGTGGTCGCTAAGGTGGAGAAGGGCAAAAGCAAGAAACTGAAATCTGTCAAGGAGCTGCTGGGTATCACAATTATGGAGAGAAGCTCTTTCGAGAAGAACCCAATCGATTTTCTGGAGGCCAAAGGTTATAAGGAAGTGAAGAAAGACCTGATCATTAAACTGCCCAAGTACAGTCTGTTTGAGCTGGAAAACGGCAGGAAACGCATGCTGGCAAGCGCTGGAGAGCTGCAGAAAGGCAATGAACTGGCCCTGCCTTCTAAGTACGTGAACTTCCTGTATCTGGCAAGCCACTACGAGAAGCTGAAAGGATCTCCAGAGGATAACGAACAGAAACAGCTGTTTGTGGAGCAGCACAAGCATTATCTGGACGAGATCATTGAACAGATTAGCGAGTTCTCTAAAAGAGTGATCCTGGCCGACGCAAATCTGGATAAGGTCCTGTCTGCTTACAACAAACACAGAGATAAGCCCATCAGGGAGCAGGCCGAAAATATCATTCATCTGTTCACTCTGACAAACCTGGGCGCACCTGCAGCTTTCAAGTACTTCGACACTACAATCGATAGAAAGAGGTACACCTCCACTAAGGAGGTGCTGGACGCTACACTGATCCATCAGAGTATTACCGGCCTGTACGAAACAAGGATTGACCTGTCTCAGCTGGGTGGCGAC**AAAAGAACTGCAGATGGATCCGAGTTCGAGAGCCCCAAGAAAAAGAGAAAGGTG**TGA**

**zABE7.10-GE**

ATGAAAAGAACTGCAGATGGATCCGAGTTCGAGAGCCCCAAGAAAAAGAGAAAGGTGATGAGCGAGGTGGAGTTTTCTCACGAGTACTGGATGAGACACGCTCTGACACTGGCCAAAAGAGCATGGGACGAGAGAGAGGTGCCTGTGGGAGCAGTGCTGGTGCACAACAACAGAGTGATCGGAGAGGGCTGGAACAGACCCATCGGAAGACACGACCCTACCGCACACGCTGAGATCATGGCCCTGAGACAGGGAGGCCTGGTGATGCAGAACTACAGACTGATCGATGCAACCCTGTACGTGACTCTGGAGCCATGTGTGATGTGCGCCGGAGCAATGATCCACAGCAGAATCGGAAGAGTGGTGTTCGGCGCTAGAGACGCCAAAACTGGAGCCGCAGGCTCTCTGATGGATGTGCTGCACCACCCCGGAATGAACCACAGAGTGGAGATCACAGAGGGCATCCTGGCTGACGAGTGTGCTGCCCTGCTGAGCGATTTCTTTAGAATGAGAAGACAGGAGATCAAGGCCCAGAAAAAGGCACAGAGCTCTACCGACTCCGGAGGATCCAGCGGAGGATCTTCCGGAAGCGAGACACCAGGCACCTCTGAGTCCGCCACTCCAGAGAGCTCTGGTGGTTCTTCTGGAGGATCTTCTGAAGTCGAATTCTCCCATGAATACTGGATGAGACACGCACTGACTCTGGCAAAAAGAGCTAGAGATGAGAGAGAGGTGCCAGTGGGCGCTGTGCTGGTGCTGAATAATAGGGTCATTGGTGAGGGCTGGAACAGAGCAATCGGACTGCACGACCCAACCGCTCACGCAGAGATCATGGCTCTGAGGCAGGGTGGACTGGTCATGCAGAATTATAGACTGATCGATGCTACACTGTACGTGACCTTCGAGCCTTGTGTGATGTGCGCAGGAGCTATGATCCACTCTAGGATCGGAAGAGTGGTGTTTGGCGTGAGAAACGCTAAGACAGGAGCAGCTGGCAGCCTGATGGACGTGCTGCACTACCCTGGTATGAATCACAGAGTGGAGATCACCGAGGGCATCCTGGCCGATGAGTGTGCCGCACTGCTGTGCTACTTCTTTAGAATGCCAAGACAGGTGTTTAACGCTCAGAAAAAGGCCCAGAGCTCTACTGATTCTGGAGGCTCCAGCGGAGGCTCTTCCGGATCCGAGACTCCTGGCACAAGCGAGTCTGCCACACCAGAGAGCTCCGGTGGCTCCAGCGGAGGCAGC**GATAAGAAGTATAGCATCGGCCTGGCTATTGGAACTAACTCCGTGGGTTGGGCAGTGATTACAGACGACTACAAGGTCCCTAGCAAGAAATTTAAGGTGCTGGGTAACACCGACAGGCACAGCATCAAGAAAAATCTGATTGGAGCCCTGCTGTTCGGTTCTGGAGAGACTGCCGAAGCAACACGCCTGAAAAGAACAGCAAGAAGGCGCTATACCAGAAGGAAGAATAGAATCTGTTACCTGCAGGAGATTTTCTCTAACGAAATGGCTAAGGTGGACGATTCATTCTTTCATAGGCTGGAGGAAAGTTTCCTGGTCGAGGAAGATAAGAAACACGAGCGCCATCCTATCTTTGGAAACATTGTGGACGAGGTCGCCTATCACGAAAAATACCCAACCATCTATCATCTGCGCAAGAAACTGGCTGACTCTACTGATAAAGCCGACCTGAGACTGATCTATCTGGCTCTGGCCCACATGATTAAGTTCAGGGGTCATTTTCTGATCGAGGGCGATCTGAACCCCGACAATTCCGATGTGGACAAGCTGTTCATCCAGCTGGTCCAGATTTACAATCAGCTGTTTGAGGAAAACCCTATTAATGCTTCCAGAGTGGACGCAAAAGCTATCCTGTCAGCCAGGCTGTCCAAGTCACGCAGACTGGAGAACCTGATTGCACAGCTGCCCGGAGAAAAGAGGAACGGTCTGTTTGGAAATCTGATCGCTCTGAGTCTGGGCCTGACTCCTAACTTCAAAAGCAATTTTGATCTGGCTGAGGACGCCAAACTGCAGCTGTCAAAGGACACATATGACGATGACCTGGATAACCTGCTGGCACAGATCGGAGATCAGTACGCTGACCTGTTCCTGGCTGCCAAAAATCTGTCCGACGCAATCCTGCTGTCAGATATTCTGAGAGTGAACAGCGAGATTACAAAAGCTCCTCTGAGTGCCAGCATGATCAAGAGATATGACGAGCACCATCAGGATCTGACCCTGCTGAAGGCTCTGGTCAGGCAGCAGCTGCCAGAGAAGTACAAGGAAATTTTCTTTGATCAGTCCAAGAACGGCTACGCCGGTTATATCGACGGAGGCGCATCACAGGAGGAATTCTACAAGTTTATCAAACCTATTCTGGAGAAGATGGACGGAACTGAGGAACTGCTGGTGAAACTGAATAGAGAGGACCTGCTGAGGAAGCAGCGCACATTTGATAACGGTTCCATCCCACACCAGATTCATCTGGGAGAGCTGCACGCTATCCTGAGGCGCCAGGAAGACTTCTACCCCTTTCTGAAAGATAACCGCGAGAAGATCGAAAAAATTCTGACCTTCAGAATCCCTTACTATGTGGGTCCACTGGCTCGCGGAAACAGCAGATTTGCCTGGATGACTCGCAAATCCGAGGAAACCATTACTCCTTGGAACTTCGAGGAAGTGGTCGATAAGGGCGCCTCTGCACAGTCCTTCATCGAGAGAATGACTAATTTTGACAAAAACCTGCCCAATGAGAAAGTGCTGCCTAAGCACTCCCTGCTGTACGAGTATTTCACTGTCTATAACGAACTGACAAAGGTGAAATACGTCACCGAGGGCATGAGAAAGCCAGCCTTCCTGTCAGGAGAGCAGAAGAAAGCAATCGTGGATCTGCTGTTTAAAACCAACAGGAAAGTGACTGTCAAGCAGCTGAAGGAGGACTACTTCAAGAAAATTGAATGCTTCGATTCCGTGGAGATCAGCGGAGTCGAAGACAGATTTAACGCAAGCCTGGGCGCTTACCACGATCTGCTGAAGATCATTAAGGATAAAGACTTCCTGGACAACGAGGAAAATGAGGATATCCTGGAAGACATTGTGCTGACACTGACCCTGTTTGAGGACAGAGGAATGATCGAGGAAAGACTGAAAACCTATGCTCATCTGTTCGATGACAAGGTGATGAAACAGCTGAAGAGAAGGCGCTACACTGGCTGGGGTAGACTGAGCAGGAAGCTGATCAACGGCATTAGGGATAAACAGTCAGGAAAGACAATCCTGGACTTTCTGAAAAGTGATGGCTTCGCCAACCGCAATTTTATGCAGCTGATTCACGATGACAGTCTGACCTTCAAAGAGGACATCCAGAAGGCTCAGGTGTCTGGACAGGGCCACTCCCTGCATGAGCAGATTGCAAACCTGGCTGGAAGCCCAGCCATCAAGAAAGGCATTCTGCAGACAGTGAAAATCGTCGATGAGCTGGTGAAAGTCATGGGCCATAAGCCCGAAAACATCGTGATTGAGATGGCTCGCGAAAATCAGACAACCCAGAAGGGTCAGAAGAACAGTAGAGAGAGGATGAAAAGAATCGAGGAAGGCATTAAGGAGCTGGGTAGCCAGATCCTGAAAGAGCACCCAGTGGAAAACACACAGCTGCAGAATGAGAAGCTGTATCTGTACTATCTGCAGAATGGAAGAGATATGTACGTGGACCAGGAGCTGGATATTAACAGGCTGTCTGATTACGACGTGGATCATATCGTCCCCCAGAGTTTCATCAAAGATGACAGCATTGACAACAAGGTGCTGACCAGGTCCGACAAAAACAGAGGAAAATCAGATAATGTCCCTAGTGAGGAAGTGGTCAAGAAAATGAAGAACTACTGGAGACAGCTGCTGAATGCCAAACTGATCACTCAGAGGAAGTTTGATAACCTGACAAAAGCAGAGCGCGGTGGACTGTCAGAACTGGACAAAGCTGGATTCATCAAGAGGCAGCTGGTGGAAACACGCCAGATCACTAAACACGTCGCACAGATTCTGGATAGTCGCATGAACACAAAGTACGATGAGAATGACAAACTGATCAGAGAAGTGAAGGTCATTACCCTGAAGAGTAAACTGGTCAGCGACTTTAGGAAAGATTTCCAGTTTTATAAGGTCCGCGAGATTAACAATTATCACCATGCCCATGACGCATACCTGAACGCCGTGGTCGGTACCGCACTGATCAAGAAATACCCAAAACTGGAGAGCGAATTCGTGTACGGAGACTATAAGGTGTACGATGTCAGAAAAATGATCGCCAAGTCCGAGCAGGAAATTGGAAAAGCTACTGCCAAGTATTTCTTTTACTCAAACATCATGAATTTCTTTAAGACAGAGATCACCCTGGCCAATGGAGAAATCCGCAAAgcGCCCCTGATTGAGACAAACGGAGAGACAGGCGAAATCGTGTGGGACAAAGGCAGAGATTTTGCAACCGTGAGGAAGGTCCTGAGCATGCCTCAAGTGAATATCGTCAAGAAAACTGAGGTGCAGACAGGCGGTTTCTCAAAAGAAAGTATTCTGCCAAAACGCAACTCTGATAAGCTGATCGCTAGAAAGAAAGACTGGGACCCTAAGAAGTATGGAGGCTTTGACTCTCCCACTGTGGCATACTCCGTCCTGGTGGTCGCTAAGGTGGAGAAGGGCAAAAGCAAGAAACTGAAATCTGTCAAGGAGCTGCTGGGTATCACAATTATGGAGAGAAGCTCTTTCGAGAAGAACCCAATCGATTTTCTGGAGGCCAAAGGTTATAAGGAAGTGAAGAAAGACCTGATCATTAAACTGCCCAAGTACAGTCTGTTTGAGCTGGAAAACGGCAGGAAACGCATGCTGGCAAGCGCTGGAGAGCTGCAGAAAGGCAATGAACTGGCCCTGCCTTCTAAGTACGTGAACTTCCTGTATCTGGCAAGCCACTACGAGAAGCTGAAAGGATCTCCAGAGGATAACGAACAGAAACAGCTGTTTGTGGAGCAGCACAAGCATTATCTGGACGAGATCATTGAACAGATTAGCGAGTTCTCTAAAAGAGTGATCCTGGCCGACGCAAATCTGGATAAGGTCCTGTCTGCTTACAACAAACACAGAGATAAGCCCATCAGGGAGCAGGCCGAAAATATCATTCATCTGTTCACTCTGACAAACCTGGGCGCACCTGCAGCTTTCAAGTACTTCGACACTACAATCGATAGAAAGAGGTACACCTCCACTAAGGAGGTGCTGGACGCTACACTGATCCATCAGAGTATTACCGGCCTGTACGAAACAAGGATTGACCTGTCTCAGCTGGGTGGCGAC**AAAAGAACTGCAGATGGATCCGAGTTCGAGAGCCCCAAGAAAAAGAGAAAGGTG**TGA**
